# 2-(2,6-Dimethoxy­phen­yl)-5-hydr­oxy-7-meth­oxy-4*H*-1-benzopyran-4-one

**DOI:** 10.1107/S1600536809032383

**Published:** 2009-08-29

**Authors:** R. Ravi Kumar, M. Krishnaiah, N. Jagadesh Kumar, D. Gunasekhar Reddy, V. G. Puranik

**Affiliations:** a Department of Physics, S. V. University, Tirupati 517 502, India; b Department of Chemistry, S. V. University, Tirupati 517 502, India; c Centre of Material Characterisation, National Chemical Laboratory, Pune 411 008, India

## Abstract

In the title compound, C_18_H_16_O_6_, the dimethoxy­phenyl ring is rotated by 61.8 (1)° from the plane of the benzopyran system. The mol­ecule is stabilized by an intra­molecular O—H⋯O hydrogen bond.

## Related literature

The title compound, along with a terpinoid and six other flavonoids, was isolated from the roots and the aerial parts of *Andrographis peniculata* Nees (Reddy *et al.*, 2003 ▶), a herb widely distributed in the plains of India and Sri Lanka (Gamble, 1956 ▶). In traditional Indian medicine, the whole plant of *A. peniculata* is extensively used in the treatment of dyspepsia, dysentery, malaria, respiratory infections and as an anti­dote for snake bites, see: Kirtikar & Basu (1975 ▶); Chopra *et al.* (1980 ▶).
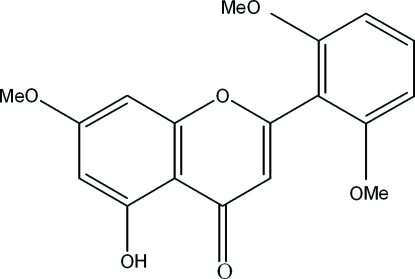


## Experimental

### 

#### Crystal data

C_18_H_16_O_6_
*M* *_r_* = 328.31
Monoclinic, 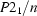

*a* = 11.003 (7) Å
*b* = 11.015 (7) Å
*c* = 13.734 (9) Å
β = 113.159 (10)°
*V* = 1530.4 (17) Å^3^
*Z* = 4
Mo *K*α radiation
μ = 0.11 mm^−1^
*T* = 295 K
0.69 × 0.37 × 0.36 mm

#### Data collection

Bruker SMART CCD area-detector diffractometer
Absorption correction: multi-scan (*SADABS*; Bruker, 2001 ▶) *T*_min_ = 0.929, *T*_max_ = 0.969
7431 measured reflections
2675 independent reflections
2300 reflections with *I* > 2σ(*I*)
*R*_int_ = 0.023

#### Refinement

*R*[*F* ^2^ > 2σ(*F* ^2^)] = 0.037
*wR*(*F* ^2^) = 0.096
*S* = 1.04
2675 reflections
221 parameters
H-atom parameters constrained
Δρ_max_ = 0.17 e Å^−3^
Δρ_min_ = −0.18 e Å^−3^

### 

Data collection: *SMART* (Bruker, 2001 ▶); cell refinement: *SAINT* (Bruker, 2002 ▶); data reduction: *SAINT*; program(s) used to solve structure: *SHELXS97* (Sheldrick, 2008 ▶); program(s) used to refine structure: *SHELXL97* (Sheldrick, 2008 ▶); molecular graphics: *ORTEP-3 for Windows* (Farrugia, 1999 ▶); software used to prepare material for publication: *enCIFer* (Allen *et al.*, 2004 ▶) and *PARST* (Nardelli, 1995 ▶).

## Supplementary Material

Crystal structure: contains datablocks I, global. DOI: 10.1107/S1600536809032383/hg2533sup1.cif

Structure factors: contains datablocks I. DOI: 10.1107/S1600536809032383/hg2533Isup2.hkl

Additional supplementary materials:  crystallographic information; 3D view; checkCIF report

## Figures and Tables

**Table 1:** Hydrogen-bond geometry (Å, °)

*D*—H⋯*A*	*D*—H	H⋯*A*	*D*⋯*A*	*D*—H⋯*A*
O6—H6⋯O4	0.82	1.82	2.560 (2)	149

**Fig. 1.**
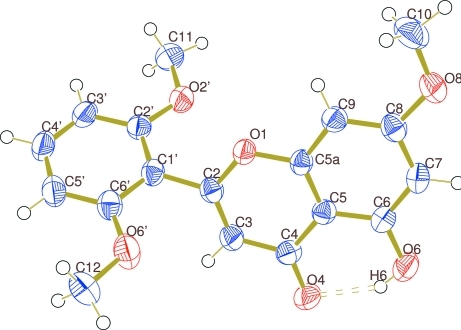
View of the molecule showing the atom-labelling scheme. Displacement ellipsoids are drawn at the 50% probability level. H atoms are represented by circles of arbitrary radius.

## supplementary crystallographic information

### Comment

The title compound along with a terpinoid and six other flavonoids were isolated from the roots and the aerial parts of Andrographis peniculata Nees (Reddy *et al.*, 2003), an erect herb widely distributed in the plains throughout India and Srilanka (Gamble, 1956). In traditional Indian medicine the whole plant of A. peniculata is extensively used in the treatment of dyspepsia, dysentery, malaria, respiratory infections and as an antidote for snake bites (Kirtikar & Basu, 1975; Chopra *et al.*, 1980). As a part of our ongoing investigation on medicinal plants, we report the structure of the title compound (I) (Fig.1).

The benzopyran ring is slightly planar with a maximum deviation from the plane of 0.034 (1) Å. The dihedral angle between the least-squares planes of the phenyl ring and the benzopyran moiety is 61.8 (1)°. The non planarity of the phenolic ring is due to the presence of the steric hindrance caused by 2',6' dioxygenation resulting the decrease in the conjugation of the phenyl ring with the carbonyl group. The absence of conjugation means that there will be more delocalization of π-electrons in C2, C3, C4, and O4 unit. The C2–C1' bond length (1.475 (2)Å) is within the 3σ of the average 4sp^2^-4sp^3^ bond distance of 1.48 Å.

### Experimental

The shade dried and powdered roots of whole plant of A. paniculata Nees (3 kg) was successively extracted with n-hexane, Me_2_CO and MeOH. The acetone extract on purification over a silica gel column using n-hexane EtoAc step gradients yielded 18 mg of the title compound with a m.p. of 196–198°C and recrystalized by slow evaporation from a hexane solution.

### Refinement

All H atoms were placed in calculated positions, with C—H = 0.93Å (aromatic H) or 0.96Å (methyl H) or 0.82Å (oxygen H) and included in the final cycles of refinement using a riding model, with *U*_iso_(H) = 1.2Ueq (C-aromatic) or *U*_iso_(H) = 1.5U_eq_ for methyl and oxygen atoms.

### Crystal data

**Table:**

C_18_H_16_O_6_	*F*(000) = 688
*M**_r_* = 328.31	*D*_x_ = 1.425 Mg m^−^^3^*D*_m_ = 1.42 Mg m^−^^3^*D*_m_ measured by none
Monoclinic, *P*2_1_/*n*	Melting point: 469 K
Hall symbol: -P 2yn	Mo *K*α radiation, λ = 0.71073 Å
*a* = 11.003 (7) Å	Cell parameters from 25 reflections
*b* = 11.015 (7) Å	θ = 2–25°
*c* = 13.734 (9) Å	µ = 0.11 mm^−^^1^
β = 113.159 (10)°	*T* = 295 K
*V* = 1530.4 (17) Å^3^	Needle, colourless
*Z* = 4	0.69 × 0.37 × 0.36 mm

### Data collection

**Table:**

Bruker SMART CCD area-detector diffractometer	2675 independent reflections
Radiation source: fine-focus sealed tube	2300 reflections with *I* > 2σ(*I*)
graphite	*R*_int_ = 0.023
Detector resolution: 0 pixels mm^-1^	θ_max_ = 25.0°, θ_min_ = 2.5°
ω scans	*h* = −7→13
Absorption correction: multi-scan (*SADABS*; Bruker, 2001)	*k* = −13→13
*T*_min_ = 0.929, *T*_max_ = 0.969	*l* = −16→16
7431 measured reflections	

### Refinement

**Table:**

Refinement on *F*^2^	Primary atom site location: structure-invariant direct methods
Least-squares matrix: full	Secondary atom site location: difference Fourier map
*R*[*F*^2^ > 2σ(*F*^2^)] = 0.037	Hydrogen site location: inferred from neighbouring sites
*wR*(*F*^2^) = 0.096	H-atom parameters constrained
*S* = 1.04	*w* = 1/[σ^2^(*F*_o_^2^) + (0.0462*P*)^2^ + 0.3516*P*] where *P* = (*F*_o_^2^ + 2*F*_c_^2^)/3
2675 reflections	(Δ/σ)_max_ < 0.001
221 parameters	Δρ_max_ = 0.17 e Å^−^^3^
0 restraints	Δρ_min_ = −0.18 e Å^−^^3^

### Special details

**Table:**

Geometry. All e.s.d.'s (except the e.s.d. in the dihedral angle between two l.s. planes) are estimated using the full covariance matrix. The cell e.s.d.'s are taken into account individually in the estimation of e.s.d.'s in distances, angles and torsion angles; correlations between e.s.d.'s in cell parameters are only used when they are defined by crystal symmetry. An approximate (isotropic) treatment of cell e.s.d.'s is used for estimating e.s.d.'s involving l.s. planes.
Refinement. Refinement of *F*^2^ against ALL reflections. The weighted *R*-factor *wR* and goodness of fit *S* are based on *F*^2^, conventional *R*-factors *R* are based on *F*, with *F* set to zero for negative *F*^2^. The threshold expression of *F*^2^ > σ(*F*^2^) is used only for calculating *R*-factors(gt) *etc*. and is ?t relevant to the choice of reflections for refinement. *R*-factors based on *F*^2^ are statistically about twice as large as those based on *F*, and *R*- factors based on ALL data will be even larger.

### Fractional atomic coordinates and isotropic or equivalent isotropic displacement parameters (Å^2^)

**Table:**

	*x*	*y*	*z*	*U*_iso_*/*U*_eq_	
O1	0.95634 (9)	0.63933 (8)	0.16154 (8)	0.0376 (3)	
C2	0.97394 (14)	0.75775 (13)	0.14376 (12)	0.0361 (3)	
C3	1.06401 (15)	0.79404 (13)	0.10773 (12)	0.0412 (4)	
H3	1.0720	0.8764	0.0967	0.049*	
C4	1.14884 (15)	0.71009 (13)	0.08555 (12)	0.0391 (3)	
O4	1.23188 (12)	0.74190 (10)	0.04933 (10)	0.0526 (3)	
C5	1.13251 (14)	0.58600 (13)	0.10870 (11)	0.0350 (3)	
C6	1.21312 (15)	0.49245 (14)	0.09621 (12)	0.0393 (3)	
O6	1.30566 (12)	0.52024 (11)	0.05872 (11)	0.0560 (3)	
H6	1.3026	0.5931	0.0458	0.084*	
C7	1.19766 (16)	0.37579 (14)	0.12164 (12)	0.0436 (4)	
H7	1.2524	0.3151	0.1146	0.052*	
C8	1.09978 (15)	0.34760 (13)	0.15815 (12)	0.0403 (3)	
O8	1.09338 (12)	0.22951 (9)	0.18118 (10)	0.0537 (3)	
C9	1.01709 (14)	0.43571 (13)	0.16945 (11)	0.0375 (3)	
H9	0.9506	0.4162	0.1926	0.045*	
C5A	1.03645 (13)	0.55351 (13)	0.14532 (11)	0.0333 (3)	
C10	0.9930 (2)	0.19296 (17)	0.21541 (17)	0.0638 (5)	
H10A	1.0044	0.2346	0.2798	0.096*	
H10B	0.9987	0.1070	0.2278	0.096*	
H10C	0.9080	0.2125	0.1618	0.096*	
C1'	0.88314 (14)	0.83990 (13)	0.16728 (12)	0.0376 (3)	
C2'	0.88837 (14)	0.85308 (13)	0.26936 (12)	0.0396 (4)	
O2'	0.97735 (11)	0.78129 (10)	0.34356 (8)	0.0467 (3)	
C3'	0.80767 (16)	0.93686 (15)	0.28990 (14)	0.0479 (4)	
H3'	0.8119	0.9467	0.3584	0.057*	
C4'	0.72169 (17)	1.00504 (15)	0.20869 (15)	0.0521 (4)	
H4'	0.6680	1.0613	0.2231	0.063*	
C5'	0.71262 (16)	0.99273 (14)	0.10730 (14)	0.0493 (4)	
H5'	0.6523	1.0388	0.0530	0.059*	
C6'	0.79435 (15)	0.91074 (14)	0.08648 (13)	0.0426 (4)	
O6'	0.79624 (13)	0.89242 (11)	−0.00985 (9)	0.0594 (3)	
C12	0.70572 (18)	0.95611 (16)	−0.09779 (14)	0.0551 (4)	
H12A	0.6172	0.9317	−0.1097	0.083*	
H12B	0.7232	0.9381	−0.1595	0.083*	
H12C	0.7152	1.0418	−0.0840	0.083*	
C11	0.99169 (19)	0.79582 (17)	0.44992 (13)	0.0557 (5)	
H11A	1.0145	0.8785	0.4713	0.084*	
H11B	1.0603	0.7430	0.4947	0.084*	
H11C	0.9100	0.7757	0.4558	0.084*	

### Atomic displacement parameters (Å^2^)

**Table:**

	*U*^11^	*U*^22^	*U*^33^	*U*^12^	*U*^13^	*U*^23^
O1	0.0400 (6)	0.0327 (5)	0.0456 (6)	0.0029 (4)	0.0227 (5)	−0.0012 (4)
C2	0.0384 (8)	0.0313 (7)	0.0373 (8)	0.0008 (6)	0.0135 (6)	−0.0020 (6)
C3	0.0455 (9)	0.0317 (8)	0.0498 (9)	0.0011 (6)	0.0223 (7)	0.0006 (7)
C4	0.0404 (8)	0.0408 (8)	0.0390 (8)	−0.0015 (7)	0.0187 (7)	−0.0003 (7)
O4	0.0583 (7)	0.0444 (6)	0.0717 (8)	−0.0006 (5)	0.0435 (7)	0.0037 (6)
C5	0.0362 (7)	0.0364 (8)	0.0322 (7)	0.0005 (6)	0.0132 (6)	−0.0025 (6)
C6	0.0388 (8)	0.0418 (8)	0.0404 (8)	0.0018 (6)	0.0188 (7)	−0.0038 (7)
O6	0.0579 (7)	0.0495 (7)	0.0795 (8)	0.0053 (6)	0.0474 (7)	0.0004 (6)
C7	0.0447 (9)	0.0378 (8)	0.0496 (9)	0.0081 (7)	0.0200 (7)	−0.0040 (7)
C8	0.0442 (8)	0.0336 (8)	0.0394 (8)	0.0001 (7)	0.0127 (7)	−0.0025 (7)
O8	0.0639 (8)	0.0317 (6)	0.0708 (8)	0.0032 (5)	0.0323 (7)	0.0043 (5)
C9	0.0387 (8)	0.0360 (8)	0.0395 (8)	−0.0017 (6)	0.0172 (7)	−0.0011 (6)
C5A	0.0340 (7)	0.0334 (7)	0.0317 (7)	0.0024 (6)	0.0120 (6)	−0.0037 (6)
C10	0.0678 (12)	0.0427 (10)	0.0818 (13)	−0.0062 (9)	0.0305 (11)	0.0124 (10)
C1'	0.0361 (8)	0.0315 (7)	0.0463 (8)	−0.0001 (6)	0.0176 (7)	−0.0041 (7)
C2'	0.0389 (8)	0.0347 (8)	0.0493 (9)	−0.0009 (6)	0.0217 (7)	0.0001 (7)
O2'	0.0522 (6)	0.0483 (7)	0.0443 (6)	0.0107 (5)	0.0242 (5)	0.0017 (5)
C3'	0.0542 (10)	0.0445 (9)	0.0574 (10)	0.0042 (8)	0.0353 (9)	−0.0015 (8)
C4'	0.0529 (10)	0.0408 (9)	0.0753 (12)	0.0102 (8)	0.0388 (10)	0.0004 (9)
C5'	0.0445 (9)	0.0408 (9)	0.0623 (11)	0.0094 (7)	0.0207 (8)	0.0033 (8)
C6'	0.0418 (8)	0.0356 (8)	0.0487 (9)	0.0010 (7)	0.0162 (7)	−0.0045 (7)
O6'	0.0695 (8)	0.0590 (8)	0.0417 (6)	0.0255 (6)	0.0131 (6)	−0.0009 (6)
C12	0.0575 (10)	0.0465 (10)	0.0509 (10)	0.0044 (8)	0.0101 (8)	0.0066 (8)
C11	0.0676 (11)	0.0585 (11)	0.0481 (10)	0.0069 (9)	0.0301 (9)	0.0014 (8)

### Geometric parameters (Å, °)

**Table:**

O1—C2	1.3549 (19)	C10—H10B	0.9600
O1—C5A	1.3692 (17)	C10—H10C	0.9600
C2—C3	1.331 (2)	C1'—C2'	1.388 (2)
C2—C1'	1.475 (2)	C1'—C6'	1.394 (2)
C3—C4	1.429 (2)	C2'—O2'	1.3555 (19)
C3—H3	0.9300	C2'—C3'	1.384 (2)
C4—O4	1.2500 (18)	O2'—C11	1.415 (2)
C4—C5	1.431 (2)	C3'—C4'	1.369 (2)
C5—C5A	1.384 (2)	C3'—H3'	0.9300
C5—C6	1.413 (2)	C4'—C5'	1.363 (3)
C6—O6	1.3445 (18)	C4'—H4'	0.9300
C6—C7	1.360 (2)	C5'—C6'	1.381 (2)
O6—H6	0.8200	C5'—H5'	0.9300
C7—C8	1.390 (2)	C6'—O6'	1.346 (2)
C7—H7	0.9300	O6'—C12	1.413 (2)
C8—O8	1.347 (2)	C12—H12A	0.9600
C8—C9	1.380 (2)	C12—H12B	0.9600
O8—C10	1.418 (2)	C12—H12C	0.9600
C9—C5A	1.376 (2)	C11—H11A	0.9600
C9—H9	0.9300	C11—H11B	0.9600
C10—H10A	0.9600	C11—H11C	0.9600
			
C2—O1—C5A	119.25 (11)	H10A—C10—H10C	109.5
C3—C2—O1	122.42 (13)	H10B—C10—H10C	109.5
C3—C2—C1'	124.37 (14)	C2'—C1'—C6'	118.90 (13)
O1—C2—C1'	113.21 (12)	C2'—C1'—C2	121.52 (13)
C2—C3—C4	121.93 (14)	C6'—C1'—C2	119.49 (13)
C2—C3—H3	119.0	O2'—C2'—C3'	124.56 (14)
C4—C3—H3	119.0	O2'—C2'—C1'	115.41 (13)
O4—C4—C3	122.98 (14)	C3'—C2'—C1'	120.03 (14)
O4—C4—C5	122.11 (13)	C2'—O2'—C11	117.66 (12)
C3—C4—C5	114.91 (13)	C4'—C3'—C2'	119.54 (15)
C5A—C5—C6	117.44 (13)	C4'—C3'—H3'	120.2
C5A—C5—C4	120.55 (13)	C2'—C3'—H3'	120.2
C6—C5—C4	122.00 (13)	C5'—C4'—C3'	121.82 (15)
O6—C6—C7	120.24 (13)	C5'—C4'—H4'	119.1
O6—C6—C5	119.10 (14)	C3'—C4'—H4'	119.1
C7—C6—C5	120.66 (14)	C4'—C5'—C6'	118.98 (16)
C6—O6—H6	109.5	C4'—C5'—H5'	120.5
C6—C7—C8	119.78 (14)	C6'—C5'—H5'	120.5
C6—C7—H7	120.1	O6'—C6'—C5'	124.35 (15)
C8—C7—H7	120.1	O6'—C6'—C1'	114.93 (13)
O8—C8—C9	123.71 (14)	C5'—C6'—C1'	120.72 (15)
O8—C8—C7	114.82 (13)	C6'—O6'—C12	119.16 (13)
C9—C8—C7	121.46 (14)	O6'—C12—H12A	109.5
C8—O8—C10	118.13 (13)	O6'—C12—H12B	109.5
C5A—C9—C8	117.69 (14)	H12A—C12—H12B	109.5
C5A—C9—H9	121.2	O6'—C12—H12C	109.5
C8—C9—H9	121.2	H12A—C12—H12C	109.5
O1—C5A—C9	116.19 (13)	H12B—C12—H12C	109.5
O1—C5A—C5	120.86 (13)	O2'—C11—H11A	109.5
C9—C5A—C5	122.94 (13)	O2'—C11—H11B	109.5
O8—C10—H10A	109.5	H11A—C11—H11B	109.5
O8—C10—H10B	109.5	O2'—C11—H11C	109.5
H10A—C10—H10B	109.5	H11A—C11—H11C	109.5
O8—C10—H10C	109.5	H11B—C11—H11C	109.5
			
C5A—O1—C2—C3	2.1 (2)	C6—C5—C5A—O1	178.64 (12)
C5A—O1—C2—C1'	−178.43 (12)	C4—C5—C5A—O1	−0.9 (2)
O1—C2—C3—C4	−0.2 (2)	C6—C5—C5A—C9	0.0 (2)
C1'—C2—C3—C4	−179.53 (14)	C4—C5—C5A—C9	−179.56 (14)
C2—C3—C4—O4	178.26 (15)	C3—C2—C1'—C2'	−115.93 (18)
C2—C3—C4—C5	−2.2 (2)	O1—C2—C1'—C2'	64.66 (18)
O4—C4—C5—C5A	−177.77 (14)	C3—C2—C1'—C6'	60.8 (2)
C3—C4—C5—C5A	2.7 (2)	O1—C2—C1'—C6'	−118.64 (15)
O4—C4—C5—C6	2.7 (2)	C6'—C1'—C2'—O2'	−179.85 (13)
C3—C4—C5—C6	−176.84 (14)	C2—C1'—C2'—O2'	−3.1 (2)
C5A—C5—C6—O6	178.63 (13)	C6'—C1'—C2'—C3'	−1.0 (2)
C4—C5—C6—O6	−1.8 (2)	C2—C1'—C2'—C3'	175.73 (14)
C5A—C5—C6—C7	−1.4 (2)	C3'—C2'—O2'—C11	−2.1 (2)
C4—C5—C6—C7	178.17 (15)	C1'—C2'—O2'—C11	176.67 (14)
O6—C6—C7—C8	−178.61 (14)	O2'—C2'—C3'—C4'	179.69 (15)
C5—C6—C7—C8	1.4 (2)	C1'—C2'—C3'—C4'	0.9 (2)
C6—C7—C8—O8	−179.87 (14)	C2'—C3'—C4'—C5'	0.2 (3)
C6—C7—C8—C9	0.0 (2)	C3'—C4'—C5'—C6'	−1.2 (3)
C9—C8—O8—C10	2.3 (2)	C4'—C5'—C6'—O6'	−178.63 (15)
C7—C8—O8—C10	−177.86 (15)	C4'—C5'—C6'—C1'	1.2 (2)
O8—C8—C9—C5A	178.53 (14)	C2'—C1'—C6'—O6'	179.76 (13)
C7—C8—C9—C5A	−1.3 (2)	C2—C1'—C6'—O6'	3.0 (2)
C2—O1—C5A—C9	177.17 (13)	C2'—C1'—C6'—C5'	−0.1 (2)
C2—O1—C5A—C5	−1.57 (19)	C2—C1'—C6'—C5'	−176.86 (14)
C8—C9—C5A—O1	−177.40 (12)	C5'—C6'—O6'—C12	−2.9 (2)
C8—C9—C5A—C5	1.3 (2)	C1'—C6'—O6'—C12	177.24 (14)

### Hydrogen-bond geometry (Å, °)

**Table:**

*D*—H···*A*	*D*—H	H···*A*	*D*···*A*	*D*—H···*A*
O6—H6···O4	0.82	1.82	2.560 (2)	149
